# ACVR1, a Therapeutic Target of Fibrodysplasia Ossificans Progressiva, Is Negatively Regulated by miR-148a

**DOI:** 10.3390/ijms13022063

**Published:** 2012-02-15

**Authors:** Hao Song, Qi Wang, Junge Wen, Shunai Liu, Xuesong Gao, Jun Cheng, Deli Zhang

**Affiliations:** 1 Institute of Infectious Diseases, Beijing Ditan Hospital, Capital Medical University, Beijing 100015, China; E-Mails: songhao@nwsuaf.edu.cn (H.S.); wangqidl04@126.com (Q.W.); liushunai@gmail.com (S.L.); gaohappay@126.com (X.G.); 2 Research Laboratory of Virology, Immunology & Bioinformatics, Northwest A & F University, Xianyang 712100, Shaanxi, China; E-Mail: wjger@163.com

**Keywords:** ACVR1, miR-148a, FOP, BMP, EndMT

## Abstract

Fibrodysplasia ossificans progressiva (FOP) is a rare congenital disorder of skeletal malformations and progressive extraskeletal ossification. There is still no effective treatment for FOP. All FOP individuals harbor conserved point mutations in *ACVR1* gene that are thought to cause ACVR1 constitutive activation and activate BMP signal pathway. The constitutively active ACVR1 is also found to be able to cause endothelial-to-mesenchymal transition (EndMT) in endothelial cells, which may cause the formation of FOP lesions. MicroRNAs (miRNAs) play an essential role in regulating cell differentiation. Here, we verified that miR-148a directly targeted the 3′ UTR of ACVR1 mRNA by reporter gene assays and mutational analysis at the miRNA binding sites, and inhibited ACVR1 both at the protein level and mRNA level. Further, we verified that miR-148a could inhibit the mRNA expression of the Inhibitor of DNA binding (Id) gene family thereby suppressing the BMP signaling pathway. This study suggests miR-148a is an important mediator of ACVR1, thus offering a new potential target for the development of therapeutic agents against FOP.

## 1. Introduction

Fibrodysplasia ossificans progressiva (FOP) is a rare and catastrophic genetic disorder of progressive heterotopic ossification (HO). It is the most catastrophic disorder of HO in humans, causing immobility through progressive metamorphosis of skeletal muscle and soft connective tissue into a second skeleton of heterotopic bone [1]. A mutation (c.G617A, p.R206H) in the glycine-serine (GS) activation domain of activin receptor type I (ACVR1, also called activin-like kinase 2 (ALK2)) was found through a linkage and sequencing analysis, and was reported in all sporadic and familial cases of classic FOP [2–6]. ACVR1 belongs to the transforming growth factor (TGF)-β superfamily, and it is a bone morphogenetic protein (BMP) type I receptor. ACVR1 is normally inactive until it binds to extracellular BMP, stimulates its phosphorylation within the GS domain, activating ACVR1 to recruit and phosphorylate Smad signaling molecules, including Smads 1, 5, and 8 within the cell; these subsequently form heteromeric complexes with the co-regulatory Smad (Smad4) and accumulate in the nucleus. Once phosphorylated, Smads then regulate target gene transcription by directly binding to DNA and interacting with different transcriptional co-activators or co-repressors [7]. The inhibitor of DNA binding (Id) genes are well-characterized BMP response genes in a variety of cell types and have broad effects on growth and differentiation [8–12]. There are four mammalian Id proteins (Id1–4) belonging to the helix-loop-helix (HLH) transcription factor family. The upregulated expression of Id1-3 in the differentiation of osteoblasts induced by BMPs, suggests a role for Id proteins in BMP-induced osteoblast lineage-specific differentiation [13]. The classic FOP-associated ACVR1 mutation R206H renders ACVR1 constitutively active and increases the phosphorylation of downstream Smad1 effector proteins. The mutant ACVR1-R206H sensitizes mesenchymal cells to BMP-induced osteoblast differentiation, and stimulates new bone formation [14]. Interestingly, it is reported that the expression of constitutively active ACVR1 in endothelial cells could cause endothelial-to-mesenchymal transition (EndMT) and acquisition of a stem cell-like phenotype. These stem-like cells could be triggered to differentiate into osteoblasts, chondrocytes or adipocytes [15]. HO in FOP lesions begins with a mesenchymal condensation, followed by chondrogenesis and endochondral ossification. It is possible that FOP lesions could be derived from EndMT.

As there is still no effective treatment for FOP, it is very urgent to find a useful therapeutic approach to inhibit BMP signaling or target ACVR1 directly.

MicroRNAs (miRNAs) are small (~22 nucleotides long), single-stranded non-coding RNAs that mediate posttranscriptional silencing of target genes. In animals, miRNAs usually bind to complementary sites in the 3′ untranslated region (UTR) of specific target genes and lead to the repression of protein expression or the promotion of target mRNA degradation [16]. miRNAs have emerged as key regulators of diverse biological processes, including cell growth and apoptosis [17], development [18], cancer [19], immune response [20] and so on. In addition, the pattern of miRNA expression in human multipotent mesenchymal stromal cells (MSC) is found to be distinct from that in pluripotent stem cells, and specific populations of miRNAs are regulated in MSC during differentiation targeted toward specific cell types [18,21]. There is increasing evidence for miRNA involvement in osteogenic differentiation of human multipotent MSCs from bone marrow [22]. A recent study validated ACVR1 as a target of miR-30c which plays a critical role in human adipocyte differentiation [23]. miRNAs have been developed as novel and promising therapeutic strategies in many diseases, such as cancer [24,25], cardiovascular diseases [26,27] and viral diseases [28,29]. Several efficient miRNA delivery methods have been developed, which make miRNA-based therapeutic products develop rapidly in recent years [24,30,31].

We initially conducted a survey of the potential miRNAs that could bind to ACVR1 mRNA using bioinformatics methods and miR-148a was identified as a strong candidate. MiR-148a belongs to miR-148 family (miR-148a, miR-148b and miR-152), which share high sequence indentity. MiR-148a is located at chromosome 7p15.2 and recent studies have found it could regulate cell proliferation [32] or promote apoptosis [33] in specific cell types. Moreover, some studies revealed that miR-148a could promote cell hypomethylation by targeting human DNMT3b [34] and DNMT1 [35], which proved to be important in aberrant CD4+ T cell DNA hypomethylation of lupus [35]. A recent study found that miR-148a expression is upregulated in dendritic cells (DCs) on maturation and activation induced by TLR agonists, which, in turn, inhibit the upregulation of MHC II expression, cytokine production, and antigen (Ag) presentation of DCs by targeting calcium/calmodulin-dependent protein kinase II (CaMKII). Therefore, miR-148a could act as negative regulator for the innate response and Ag-presenting capacity of DC [36]. Furthermore, miR-148a may play a role in metastasis related to hepatocellular carcinoma [37]. Interestingly, it was found that miR-148a was both underexpressed in MSCs that differentiate from human embryonic stem cells (ESs) (called ES-MSCs) compared with normal ESs [38], and in osteo-differentiated MSCs compared with normal MSCs [22]. We speculated that the negative correlation between the downregulation of miR-148a and upregulation of ACVR1 may reveal a mechanism. In this study, we found miR-148a could negatively regulate ACVR1 directly by targeting the 3′ UTR of ACVR1 mRNA.

## 2. Results and Discussion

### 2.1. Interaction Between miR-148a and ACVR1 3′ UTR Binding Sites

**Figure 1:**
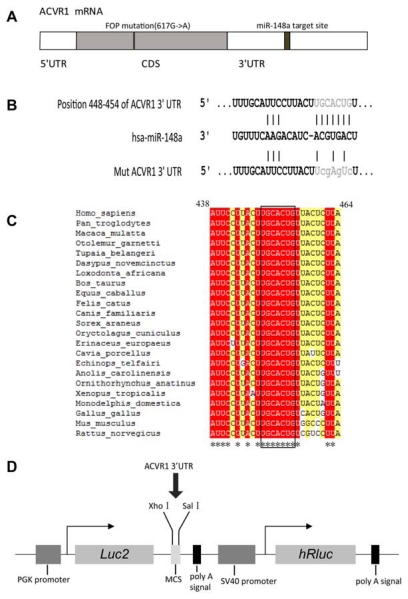
Interaction of miR-148a with the 3′ UTR of ACVR1 mRNA. (**A**) Schema of human ACVR1 mRNA and miR-148a binding site in its 3′-UTR sequence. (**B**) Schematic indication of miR-148a binding sites in the 3′ UTR of ACVR1 mRNA based on TargetScan 5.1 prediction (seed sequences at 448–454 bp of ACVR1 3′ UTR were highlighted in grey, mutant bases for vector pmir-ACVR1-3′UTR-mut construction were shown as lowercase letters). (**C**) Multiple sequence alignment of the miR-148a binding sites in different species. The alignment was performed with ClustalW. The miR-148a seed sequences at 448–454 bp of ACVR1 3′ UTR were framed. * conservative site. (**D**) Luciferase reporter vector structure. The vector contained two expression units; one for the firefly luciferase gene (luc2) and the other for the Renilla luciferase gene (hRluc-neo fusion) expression. Luc2 unit was driven by a human phosphoglycerate kinase (PGK) promoter and contained multiple cloning sites (MCS) downstream of the luc2 sequence. ACVR1 3′ UTR containing a putative miRNA target region was cloned into the MCS. The Renilla luciferase unit was driven by an SV40 early promoter and used to adjust for variations in transfection efficiency among experiments and normalize firefly luciferase activity.

In initial studies, TargetScan 5.1 [39], miRanda [40] and miRDB [41] were used to identify miRNAs with putative binding sites in ACVR1 3′ UTR. There were 33 miRNAs (miRNA list is shown in Supplementary Table S1) including miR-148a could be predicted by all the three programs. As miR-148a had been shown to be related with osteo-differentiation [22,38], we chose miR-148a for further study. The predicted interaction between miR-148a and its target sites in ACVR1 3′ UTR is illustrated in Figure 1B. We found that the miR-148a binding sites were conserved in different vertebrates (Figure 1C).

**Figure 2:**
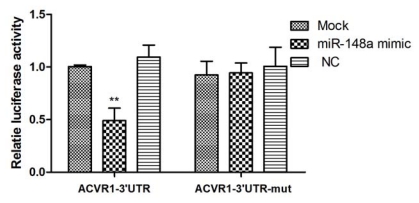
miR-148a directly targets human ACVR1 3′ UTR. Relative luciferase activity derived from pmirGLO-ACVR1-3′UTR or pmirGLO-ACVR1-3′UTR-mut were co-transfected with miR-148a mimic or NC mimic in HeLa cells. The transfection of pmirGLO-ACVR1-3′UTR or pmirGLO-ACVR1-3′UTR-mut alone was used as control (Mock). Firefly and Renilla luciferase activities were determined, and firefly luciferase was normalized to Renilla luciferase activity. Results are expressed as relative activities against the activity of mock mimic transfection. ** *p* < 0.01.

To test whether miR-148a can alter the expression of ACVR1, we cloned a region of its 3′ UTR containing the putative miRNA-binding sequences into a dual-luciferase miRNA target expression vector (pmirGLO vector). The structure of the luciferase reporter vector is illustrated in Figure 1D. We co-transfected pmirGLO-ACVR1-3′UTR and miR-148a mimic into HeLa cells. Negative control (NC) mimic and mock transfection were assayed at the same time. The presence of the interaction between miRNA and mRNA would reduce the firefly luciferase activity normalized to the Renilla luciferase activity of the control transfection. In the presence of miR-148a mimic, the constructs bearing the 3′ UTR of ACVR1 mRNA showed decreases in luciferase activity of 50% compared with the control (Figure 2).

To further investigate whether the putative target site was regulated by miR-148a, we introduced point mutations to the corresponding seed sequence at pmirGLO-ACVR1-3′UTR to eliminate the predicted binding by miR-148a. As shown in Figure 2, suppression of the reporter activity by miR-148a was almost fully relieved by mutation of the conserved seed complementary site, denoting that the matching site identified strongly contribute to the miRNA:mRNA interaction that mediates the post-transcriptional inhibition of the ACVR1 expression.

### 2.2. Endogenous Target Post-Transcriptional Repression by miR-148a

**Figure 3:**
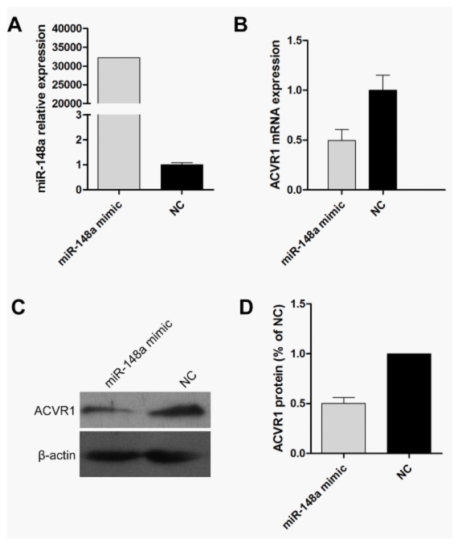
Verification of target genes of miR-148a. (**A**) QRT-PCR results of miR-148a expression level in HeLa cells transfected with miR-148a mimic or NC mimic for 48 h. RNU44 (RNA, U44 small nuclear) was used as the normalization control. (**B**) QRT-PCR results of mRNA level of ACVR1 in HeLa cells transfected as described in A. GAPDH was used as the normalization control. (**C**) Western blot analysis of ACVR1 protein level in HeLa cells transfected as described in A for 72 h. (**D**) The bands’ intensity in C is quantified using the ImageJ 1.43 software [42]. The relative intensity against β-actin was calculated, and fold change relative to the relative intensity in transfected NC cells is presented.

To investigate the effects of miR-148a on the endogenous expression of its targets and in order to obtain high miR-148a expression levels, we transiently transfected miR-148a mimic into HeLa cells, and a NC mimic was used as a control. After transfection (48 h or 72 h), the expression of miR-148a was tested by quantitative Real time PCR (qRT-PCR) and the mRNA or protein expression of ACVR1 was determined by qRT-PCR or western blot analysis. Obviously, the expression of miR-148a was increasing dramatically compared with negative control samples (Figure 3A). In HeLa cell line, both mRNA and protein levels of ACVR1 were significantly reduced by miR-148a overexpression compared with negative controls (Figure 3B–D). The results showed that enforced expression of miR-148a led to a significant decrease in endogenous ACVR1 mRNA and protein, suggesting that the endogenous expression of ACVR1 was down regulated by miR-148a.

### 2.3. Inhibition of BMP Signaling by miR-148a

**Figure 4:**
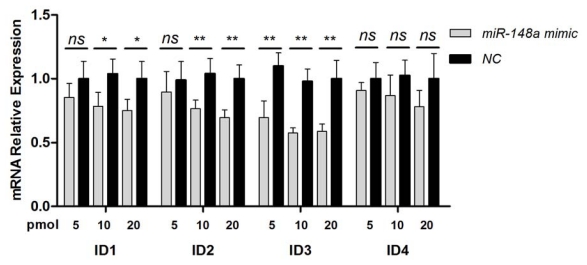
miR-148a inhibition of the BMP signaling pathway detected as reduced expression of Id1–3 mRNA levels. The mRNA levels of Id1–4 were detected by qRT-PCR in HeLa cells transfected with 5, 10,or 20 pmol miR-148a mimic or NC mimic for 48 h. * *p* < 0.05, ** *p* < 0.01, ^ns^ *p* > 0.05.

To investigate whether the inhibition of ACVR1 by miR-148a could influence the BMP signaling pathway, we examined the endogenous mRNA levels of Id1-4 when miR-148a was overexpressed in HeLa cells. QRT-PCR of transfected HeLa cells showed that miR-148a overexpression significantly decreased the mRNA expression of Id1, Id2 and Id3 in a dose-dependent way (Figure 4). In contrast, the expressions of Id4 mRNA had no significant difference between two groups. Id4 mRNA was detected at very low abundance with high ct values in qRT-PCR assay.

HeLa cells have previously been reported to have an indirect osteogenic effect by secreting BMP4 and BMP6 continuously to the surrounding host cells when implanted in a mouse model of heterotopic bone formation [43–45]. HeLa cells have been shown to be strongly positive for alkaline phosphatase (ALP) activity, with high expressing levels of several ALP isoforms [46,47]. In addition, HeLa cells have been reported to deposit mineral in osteogenic medium treated cell cultures and express osteocalcin, which is an established osteogenic marker [47–49]. The osteogenic properties of HeLa cell line make it suitable for BMP signaling pathway research. Ids are key component of the BMP signaling pathway [13]. The mRNA and protein expression of Ids could be dectected in HeLa cells except Id4 [50,51]. The decreased mRNA expression of Id1–3 indicated that the BMP signaling was inhibited after miR-148a overexpression.

miRNAs are of ever increasing importance as post-transcriptional regulators of gene expression following transcription. They have been demonstrated to play a significant role in osteogenic differentiation by altering expression of important genes in different pathways [52–54]. In this study, we have confirmed by multiple methods that miR-148a regulated the expression of ACVR1 by directly targeting the 3′ UTR of its mRNA, revealing a mechanism of post-transcriptional regulation of ACVR1 expression.

ACVR1 is a very important protein in TGF-β and BMP pathway. The constitutively activating mutation (c.G617A, p.R206H) of ACVR1 dysregulates BMP signaling and initiates the formation of a second skeleton of heterotopic bone [2–6]. It is reported that ACVR1 is also very important in EndMT [15]. In our knowledge, there is still no effective inhibitor for ACVR1. miRNAs mediated post-transcription silence has been used in therapy [24,55]. We found miR-148a could directly regulate the expression of ACVR1 both at mRNA and protein level by binding to the complementary sites in the 3′ UTR of ACVR1 mRNA. The mutations or single nucleotide polymorphisms in miRNA binding sites could influence normal miRNA mechanisms, which may cause diseases [56–58] or phenotypic variation [59]. However, in this study we found the miR-148a binding sites were conserved in different vertebrates. So we speculate that miR-148a could be used to inhibit the constitutive activity mutated ACVR1 as it targets the 3′ UTR of ACVR1 mRNA, regardless of the mutation in the translated region. In addition, as activated ACVR1 is very important for EndMT, miR-148a inhibitor may be useful to activate ACVR1 expression or enhance its expression. However, it should be noted that this study has been examined only in HeLa cells. Further researches were needed to further validate the effect of miR-148a on other types of cells especially endothelial cells. Despite its preliminary character, this study can clearly indicate that ACVR1 was a target of miR-148a, thus may provide a new approach for FOP therapy in the future.

## 3. Experimental Section

### 3.1. Cell Culture

HeLa cells and A549 cell lines were cultured in RPMI medium 1640 supplemented with 10% fetal bovine serum, 100 units/mL penicillin and 100 units/mL streptomycin in a humidified chamber containing 5% CO_2_ at 37 °C.

### 3.2. Plasmid Construction

Genomic DNA was isolated from A549 cells using a Genomic DNA Purification kit (Promega, USA). A 1 kb fragment(+1~+1001, chromosome 2: 158,593,042–158,594,042) of 3′ UTR (total 1102 bp) of human *ACVR1* gene was amplified using Pfu polymerase and the following primer set was designed according to the reference sequence of human ACVR1 mRNA(NM_001111067): ACVR1-3′UTR-F: 5′-CCTCGAGCATTTTCATAGTGTCAAGAAGGAAGA-3′ (*Xho*I); ACVR1-3′UTR-R: 5′-AGTCGACTCTGGCAGAGTTTAAATGCACGTAAT-3′ (*Sal*I). The obtained DNA fragment was inserted into the pmirGLO Vector (Promega, USA). The resulting vector was sequence-verified and named pmir-ACVR1-3′UTR.

### 3.3. miRNA Targets Prediction

In this study, TargetScan 5.1 [39], miRanda [40] and miRDB [41] were used for identification of miRNA target sites.

### 3.4. Site-Directed Mutagenesis

The vector pmir-ACVR1-3′UTR was utilized to perform site-directed mutagenesis of the putative miR-148a binding sites following the quick change site-directed mutagenesis protocol (Stratagene, USA). The mutant bases in putative miR-148a seed sequence were shown in Figure 1B. The oligonucleotides incorporating mutant bases were as follows: 5′-CAAGTTGGGTCTTTAAAA TTAAGAGTAAGACTCGAAGTAAGGAATGCAAAAATTCCTAGTG-3′. The PCR reaction was performed for 30 cycles (95 °C for 30 s, 55 °C for 30 s, 68 °C for 10 min) following an initial denaturation at 95 °C for 30 s. The mixture of input and amplified DNA was digested directly with *Dpn*I and then transformed into XL-10 Blue cells. The nucleotide sequences of the mutant were confirmed by sequencing, and the resulting vector was named pmir-ACVR1-3′UTR-mut.

### 3.5. miRNAs and Transfection

The hsa-miR-148a (MI0000253) mimic and NC mimic (5′-UUCUCCGAACGUGUCACGUTT-3′) were synthesized by GenePharma (Shanghai, China). The miRNA transfection was performed using Lipofectamine 2000 (Invitrogen, Carlsbad, CA, USA) according to the manufacturer's instructions. In brief, cells were plated in 6-well plate to 40%–60% confluence. For each well, 100 pmol miR-148a mimic or NC was added. Total RNA or protein were prepared 48 h or 72 h after transfection and were used for qRT-PCR or western blot analysis. To study the potential effect of miR-148a on BMP signaling pathway, cells were plated in 24-well plates and transfected with 5, 10 or 20 pmol of miR-148a mimic or NC using Lipofectamine 2000. The cells were harvested at 48 h post-transfection for qRT-PCR analysis.

### 3.6. RNA Isolation and Real-Time PCR Analysis

Total RNA was isolated using the mirVana miRNA PARIS kit (Ambion), in accordance with the manufacture’s protocol. A total of 0.5 μg of RNA from each sample was used to generate cDNA by reverse transcription with the PrimeScript RT reagent Kit (Takara, Japan). Using the resulting cDNAs as templates, the gene expression levels were measured using an ABI Prism 7500 (Applied Biosystems, USA) and SYBR^®^ Premix Ex Taq™ II (Takara, Japan) according to the manufacturer’s instructions. GAPDH was used as reference for normalizing data. The primer sequences for measurement of mRNA expression were: 5′-TGGAGAAACCTGCCAAGTATG-3′ (GAPDH-forward); 5′-GGAGACAACCT GGTCCTCAG-3′ (GAPDH-reverse); 5′-GCAACCAAGAACGCCTCAATC-3′ (ACVR1-forward); 5′-TTTCCCGACACACTCCAACAG-3′(ACVR1-reverse). 5′-TACATCAGGGACCTTCAGTTGGAG-3′ (Id1-forward); 5′-TTCAGCGACACAAGATGCGATC-3′ (Id1-reverse); 5′-TGTGGCTGAATAA GCGGTGTTC-3′ (Id2-forward); 5′-TCCATTCAACTTGTCCTCCTTGTG-3′ (Id2-reverse); 5′-CCCACCTTCCCATCCAGACAG-3′ (Id3-forward); 5′-TGGGCAGGGCGAAGTTGG-3′ (Id3-reverse); 5′-AACAAGCAGGGCGACAGCATTC-3′ (Id4-forward); 5′-GCTCCTGGCTCGGGCTCAG-3′ (Id4-reverse).

Quantitative RT-PCR of miRNAs was performed using Taqman MicroRNA assays (Applied Biosystems, USA) according to the manufacturer’s instructions with the 7500 real-time RT-PCR system. The expression level of the small nuclear RNU44 was used as the normalization control. All assays were performed in quadruplicate. The relative expression levels were calculated using the 2^−ΔΔCt^ method.

### 3.7. Luciferase Reporter Assay

HeLa cells were seeded in 48-well plate, grown to a density of 80%–90% confluence and then co-transfected to each well with 150 ng of luciferase reporter vector and 10 pmol miRNAmimic using Lipofectamine 2000 (Invitrogen, Carlsbad, CA, USA) according to the manufacturer’s protocol. Cells were harvested and lysed in 50 μL of passive lysis buffer 24 h post-transfection. A fraction of protein was subjected to a dual-luciferase reporter assay system (Promega, USA). Firefly luciferase activity and Renilla luciferase activity were measured sequentially by a Veritas Microplate Luminometer (Turner BioSystems, USA). Firefly luciferase activity was normalized to Renilla luciferase activity. All transfections were performed in triplicate, and the luciferase activities were expressed as the mean ± SD of three independent experiments.

### 3.8. Western Blot

After transfection, the cells were collected and washed with cold PBS three times. The PBS was decanted, and the cell pellet was resuspended in 100 μL of lysis buffer (Fermentas, Canada) for 10 min on ice. The resulting solution was then combined with 25 μL 5× SDS sample buffer and boiled for 10 min. The samples were resolved on a 10% SDS-PAGE gel and transferred to PVDF membranes (Millipore, USA). The membranes were blocked using 5% nonfat dry milk for 1 h at room temperature. The filters were incubated overnight with primary antibodies at 4 °C and then washed. After further incubation with a secondary antibody conjugated with HRP for 1 h at room temperature, the membranes were washed extensively, and the signals were developed by Immun-starHRP Chemiluminescent Kit (Bio-Rad, USA). Western blots were quantified using the ImageJ 1.43 software (National Institutes of Health, Bethesda, MD) after densitometric scanning of the films. Antibodies directed against ACVR1(sc-25449) and β-actin (sc-47778) were purchased from Santa Cruz Biotechnology (Santa Cruz, USA); goat anti-mouse(GAM)-HRP conjugate (# 170-5047) and goat anti-rabbit (GAR)-HRP conjugate (# 170-5046) were purchased from Bio-Rad. β-actin was used as a loading control.

### 3.9. Statistical Analysis

Data was expressed using the mean and the standard deviation when at least three independent experiments were performed. Statistical analysis was performed using the two-tailed student’s *t*-test, and *p* < 0.05 was considered of significant difference.

## 4. Conclusions

We demonstrated that miR-148a could regulate the expression of ACVR1 by directly targeting the 3′ UTR of its mRNA and modulate the BMP signaling pathway, which suggest that miR-148a could be used as a new target for the development of therapeutic agents against FOP.

## Supplementary Materials
