# Supplementary material for: ACVR1, a Therapeutic Target of Fibrodysplasia Ossificans Progressiva, Is Negatively Regulated by miR-148a
Source: Int J Mol Sci. 2012 Feb 15;13(2):2063–77. doi: 10.3390/ijms13022063 (PMC3292007; doi:10.3390/ijms13022063)
Supplement: Supplementary file 1 [file ijms-13-02063-s001.pdf]

Supplementary Materials

Table S1. The miRNA list that could potentially target ACVR1 3' UTR. Targetscan, miRanda and miRDB were used for prediction and the miRNAs that could be predicted by all the three programs are shown below.

MicroRNA name
hsa-miR-130a
hsa-miR-130b
hsa-miR-137
hsa-miR-148a
hsa-miR-148b
hsa-miR-152
hsa-miR-182
hsa-miR-193a-5p
hsa-miR-197
hsa-miR-220b
hsa-miR-301a
hsa-miR-301b
hsa-miR-30a
hsa-miR-30b
hsa-miR-30c
hsa-miR-30d
hsa-miR-30e
hsa-miR-330-3p
hsa-miR-365
hsa-miR-384
hsa-miR-448
hsa-miR-450b-5p
hsa-miR-454
hsa-miR-518a-5p
hsa-miR-519a
hsa-miR-519b-3p
hsa-miR-519c-3p
hsa-miR-527
hsa-miR-548n
hsa-miR-568
hsa-miR-586
hsa-miR-606
hsa-miR-651

© 2012 by the authors; licensee MDPI, Basel, Switzerland. This article is an open access article distributed under the terms and conditions of the Creative Commons Attribution license (http://creativecommons.org/licenses/by/3.0/).
